# The Vip3Ag4 Insecticidal Protoxin from *Bacillus thuringiensis* Adopts A Tetrameric Configuration That Is Maintained on Proteolysis

**DOI:** 10.3390/toxins9050165

**Published:** 2017-05-14

**Authors:** Leopoldo Palma, David J. Scott, Gemma Harris, Salah-Ud Din, Thomas L. Williams, Oliver J. Roberts, Mark T. Young, Primitivo Caballero, Colin Berry

**Affiliations:** 1 Instituto de Agrobiotecnología, CSIC-UPNA-Gobierno de Navarra, Campus Arrosadía, Mutilva 31192, Navarra, Spain; lpalma.leopoldo@gmail.com (L.P.); pcm92@unavarra.es (P.C.); 2 School of Biosciences, University of Nottingham, Sutton Bonnington Campus, Leicestershire LE12 5RD, UK; david.scott@nottingham.ac.uk; 3 Research Complex at Harwell, Rutherford Appleton Laboratory, Harwell Campus, Oxfordshire OX11 0FA, UK; gemma.harris@rc-harwell.ac.uk; 4 ISIS Spallation Neutron and Muon Source, Rutherford Appleton Laboratory, Harwell Campus, Oxfordshire OX11 0QX, UK; 5 Cardiff School of Biosciences, Cardiff University, Park Place, Cardiff CF10 3AT, UK; salahuddin@cemb.edu.pk (S.-U.D.); o.roberts1@nhs.net (O.J.R.); youngmt@cardiff.ac.uk (M.T.Y.); 6 Cardiff School of Chemistry, Cardiff University, Park Place, Cardiff CF10 3AT, UK; williamst30@cardiff.ac.uk

**Keywords:** Vip3 toxin, electron microscopy, surface topology

## Abstract

The Vip3 proteins produced during vegetative growth by strains of the bacterium *Bacillus thuringiensis* show insecticidal activity against lepidopteran insects with a mechanism of action that may involve pore formation and apoptosis. These proteins are promising supplements to our arsenal of insecticidal proteins, but the molecular details of their activity are not understood. As a first step in the structural characterisation of these proteins, we have analysed their secondary structure and resolved the surface topology of a tetrameric complex of the Vip3Ag4 protein by transmission electron microscopy. Sites sensitive to proteolysis by trypsin are identified and the trypsin-cleaved protein appears to retain a similar structure as an octomeric complex comprising four copies each of the ~65 kDa and ~21 kDa products of proteolysis. This processed form of the toxin may represent the active toxin. The quality and monodispersity of the protein produced in this study make Vip3Ag4 a candidate for more detailed structural analysis using cryo-electron microscopy.

## 1. Introduction

*Bacillus thuringiensis* (Bt) is a Gram-positive entomopathogenic bacterium with strains that show toxicity to a wide variety of invertebrates [1]. The best-studied toxins produced by these strains are the crystal (Cry) and cytolytic (Cyt) toxins, also known as δ‑endotoxins, which are synthesized during the stationary growth phase and into sporulation as parasporal crystalline inclusions [2]. In addition, Bt synthesizes other insecticidal proteins that are secreted to the culture medium during the vegetative growth phase: vegetative insecticidal proteins (Vip) [3,4] and secreted insecticidal proteins (Sip) [5]. Vip proteins have been classified into four families; Vip1, Vip2, Vip3 and Vip4, according to their degree of amino acid similarity [6]. Vip1 and Vip2 act together as a binary toxin with insecticidal activity against some coleopteran [4] and hemipteran pests [7] and function through the ADP-ribosyltransferase activity of Vip2 [8], the structure of which has been solved [9]. The Vip4 protein is distantly related to the Vip1 component of this binary toxin, but its activity remains unknown to date [10].

Vip3 proteins have no primary sequence homology to the other Vip proteins or other toxins and exhibit toxicity against lepidopteran larvae [3,11]. As for the Cry and Cyt toxins of Bt, the Vip3 proteins are named according to the degree of amino acid identity between family members with subdivisions of the protein family having different secondary rank (denoted by the capital letter) at <78% identity, tertiary rank (denoted by the lower case letter) at <95%, and a quaternary rank (denoted by the final number) assigned to each new entry into the nomenclature [6]. Vip3Aa proteins appear to recognise distinct receptors from Cry1 toxins in *Manduca sexta* [12]*, Agrotis segetum* [13] and *Spodoptera littoralis* [14], which is consistent with reports that insects resistant to Cry toxins are not cross-resistant to Vip3 proteins [12,15,16]. This has made Vip3 proteins attractive as additional insect resistance traits in transgenic crops (e.g., [17,18]). 

The current understanding of the mode of action of Vip3 proteins remains limited, although a number of steps towards intoxication are known [19]. Proteolysis of the ~88 kDa full-length Vip3A proteins to ~65 kDa by trypsin or the gut juices of both susceptible and non-susceptible insects has been reported [12,13,20,21]. It has been proposed that differences in the amounts of further digestion products accumulated may be linked to levels of susceptibility to the toxins [20,21]. Binding of proteolytically processed Vip3A proteins to ligands of 55 and 100 kDa in *Ephestia kuehniella* [14], 80 and 100 kDa in *Manduca sexta* [12] or of 65 kDa in *Agrotis segetum* [13] has been reported and using a two‑hybrid system, a putative ~43 kDa receptor with homology with the tenacins has been identified in *Agrotis ipsilon* [22]. Toxin activated by gut juices is able to form pores in planar lipid bilayers and dissected *Manduca sexta* gut tissue [12]. The histopathology of intoxication shows cytoplasmic vacuolization and breakup of the brush border membrane [13,14] and there is evidence that Vip3Aa causes apoptosis in *Spodoptera frugiperda* Sf9 cells [22,23]. However, an understanding of the molecular mechanism of the Vip3 proteins is hampered by the absence of structural data. As a first stage in the process of 3D-structure determination, here we describe the expression, purification and analysis of the trypsin processing of the Vip3Ag4 protein. We analyse its secondary structure and present approximately 33 Å resolution surface topologies of both a Vip3Ag4 tetramer and a trypsin-processed complex, determined via transmission electron microscopy and single particle analysis.

## 2. Results and Discussion

### 2.1. Purification of Vip3Ag4

**Figure 1:**
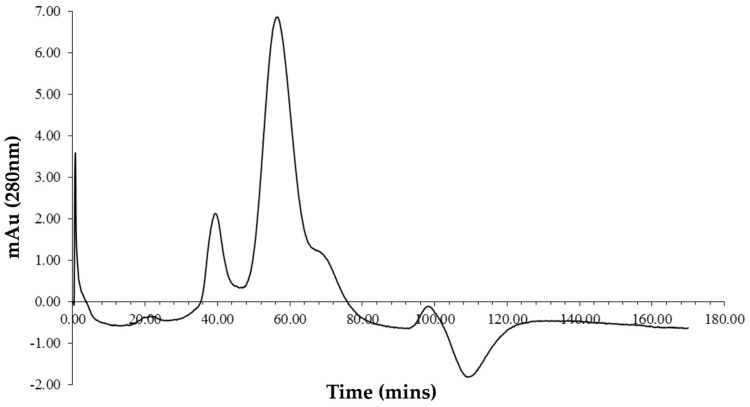
Size exclusion chromatography analysis of the initial Vip3Ag4 preparation. The absorption of the Vip3Ag4 at 280 nm over time is shown. The major peak, emerging around 60 min, corresponds to the Vip3 tetramer.

Expression of Vip3Ag4 in *Escherichia coli* and purification by nickel affinity chromatography resulted in a single band of around 91 kDa on SDS PAGE, consistent with the expected size for the recombinant protein including His-tag (91.38 kDa). Size exclusion chromatography produced several peaks (Figure 1). The first, small peak emerging at 40 min may represent aggregated material, the largest peak (60 min) has an approximate molecular mass (calculated with respect to gel filtration standards) of 354 kDa, which corresponds approximately to a tetrameric form. There is a further shoulder to this peak (~70 min) that appears to represent monomeric Vip3Ag4. A recent study with Vip3Aa35 (82% identical to Vip3Ag4), activated with trypsin, indicated the presence of aggregated, monomeric and tetrameric forms of this protein; the proportions of these forms could be manipulated by changing buffer conditions [24]. Fractions corresponding to the putative tetrameric form of Vip3Ag4, chosen to exclude those that might include the monomeric form were combined and the protein was concentrated to 1 mg/mL. Mass spectrometric analysis of this material revealed a protein of 91,245.5 Da, a size that demonstrates the production of the monomeric, His-tagged protein, lacking the initial Met residue (theoretical mass 91,245.7). Initiator methionine residues are often removed by *E. coli* methionine aminopeptidases, especially when, as in the recombinant Vip3Ag4, the next residue has a small sidechain (in this case glycine) [25].

### 2.2. Trypsin Processin

Trypsin digestion of the Vip3Ag4 protein produced bands of ~65 kDa and ~21 kDa on SDS PAGE. The larger band was subjected to five cycles of N‑terminal sequencing and revealed the sequence N/K-S-S-E/P-A, which is consistent with the sequence NSSPA that starts at residue N199 of the native Vip3Ag4 sequence (residue 233 of the His-tagged recombinant protein). Mass spectrometry of the products of trypsin digestion revealed two major peaks with molecular weights of 65,401.0 and 20,740.0. These masses match the region of Vip3Ag4 from N199 to the C—terminal residue (expected mass 65,401.2) and the region from D33 of the His tagged recombinant protein (two residues upstream of the initiator Met of the naturally-occurring protein) to K182 of the native Vip3Ag4 sequence (residue 216 of the recombinant protein—expected mass 20,740.7). Cleavage sites are shown on the primary sequence of the recombinant Vip3Ag4 in Figure S1. The trypsin processing indicated by these peaks would also produce a further fragment from F183 to K198 (FEDLTFATETTLKVKK) and the N‑terminal region of the recombinant protein, but peaks corresponding to these fragments were not observed. The pattern of digestion seen with Vip3Ag4 is consistent with other reports of Vip3 processing by both trypsin and insect gut extracts [12,13,20,21]. Estruch et al. [22] reported a similar initial processing of Vip3Aa by gut juices from *A. ipsilon* to fragments of ~22 kDa (residues 1–198) and ~65 kDa (residues 200–789), although they did not see processing after the conserved K182 residue. Gut juice was then seen to cause further proteolysis of the ~65 kDa Vip3Aa fragment to ~45 kDa (residues 412–789) or ~33 kDa (residues 200–455). These further degradation products were not seen in the present study with Vip3Ag4. This may be due to sequence differences in the two toxins: Vip3Ag4 has the sequence KTK (residues 455–457) in place of the Vip3Aa sequence KKK, which may affect trypsin-like cleavage in this region to generate the ~33 kDa product. The presence of proteolytic activities other than trypsin in the gut juice is also likely given the cleavage between Thr411 and Asn412 that generates the ~45 kDa product [22], since this site is conserved in Vip3Ag4. 

**Figure 2:**
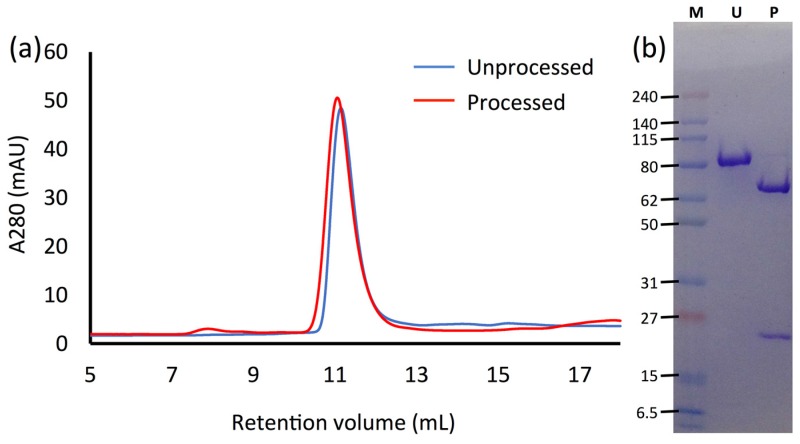
Analysis of purified Vip3Ag4 before and after trypsin treatment. (**a**) SEC of unprocessed and trypsin-treated recombinant Vip3Ag4; (**b**) SDS Poly Acrylamide Gel Electrophoresis of unprocessed Vip3Ag4 (U) and the SEC peak from trypsin-treated Vip3Ag4 (P); the sizes of protein markers (M) are indicated.

When the trypsin-treated Vip3Ag was analysed by Size exclusion chromatography (SEC), there was no significant change in the elution time for the major peak compared to unprocessed material (Figure 2a) and when fractions from this peak were analysed by SDS PAGE, both ~21 and ~65 kDa bands were seen (Figure 2b). This indicates not only that the ~21 kDa trypsin-cleaved fragment from D33-K216 of the recombinant protein remains associated with the larger ~65 kDa fragment after digestion, but also that the processed Vip3Ag toxin remains in the multimeric complex form in solution. Kunthic et al. recently described a variety of monomeric and oligomeric forms of trypsin-treated Vip3Aa35 including a form arising after the addition and dialysis of detergent that appeared to be a tetramer of the ~66 kDa polypeptide only. Our experiments suggest that, in the absence of detergent treatment, Vip3Ag4 treated with trypsin is an octameric complex consisting of tetramers of both the ~65 kDa and 21 kDa fragments. This is consistent with the finding that the trypsin cleavage products of Vip3Aa16 (82% identical to Vip3Ag4) remain associated [26] and may indicate that the proteolytically cleaved complex is the active form of the toxin. The sensitivity of *S. exigua* larvae to Vip35Aa in full-length or trypsin-processed forms shows no significant difference [24]. Since trypsin-like cleavage of the full-length form would be expected in vivo in the insect gut, these results show that trypsin cleavage does not reduce activity and the processed form may be the active agent. The stability of the 65 kDa product of Vip3Aa shows a correlation to differences in species susceptibility [20] and gut juice-processed Vip3A is able to form pores, whereas full-length protein could not [12]. 

### 2.3. Circular Dichroism

To estimate the secondary structure content of the Vip3Ag4 protein, circular dichroism analysis was performed. Analysis of the data using the Dichroweb server found the best fit of data to reference datasets using the CONTIN analysis program and reference set 3 [27]. Using these tools, a good match with reference data was obtained for most of the curve (Figure S2), suggesting a secondary structure content in the tetrameric Vip3Ag4 of ~11% alpha helix, ~38% ß sheet, 22% loops and 30% unordered. 

### 2.4. SEC-MALLS

In order to achieve an accurate reconstruction of TEM data to produce a structure, it is essential to know the multimeric form of the protein being analysed. To verify our initial observations during purification, we undertook SEC-MALLS and analytical ultracentrifugation analyses.

**Figure 3:**
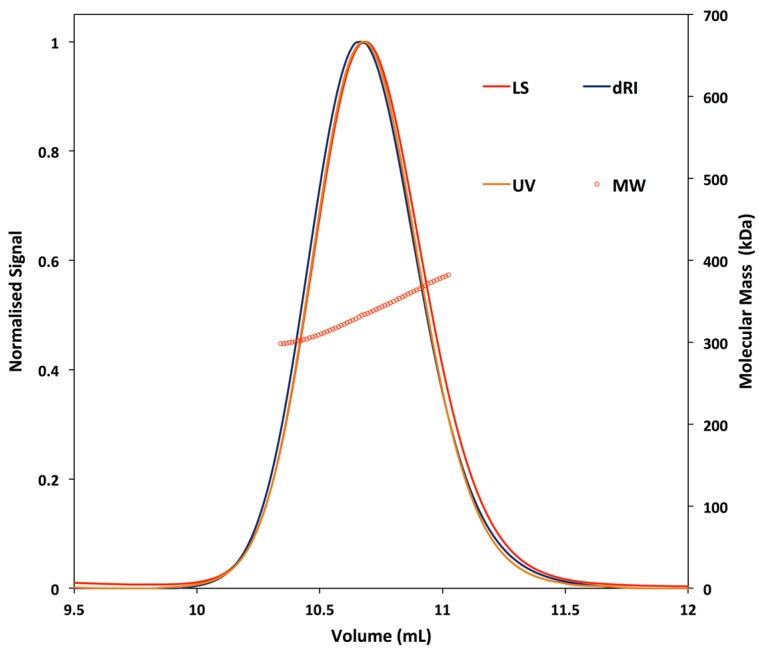
SEC-MALLS analysis of the oligomeric state of Vip3Ag4. LS—light scattering; dRI—differential refractive index; UV—UV at 280 nm; MW—molecular weight.

The elution profile of the Vip3Ag4 sample contained a single peak (Figure 3). The molecular mass obtained from the SEC-MALLS analysis was consistent with that of a Vip3Ag4 tetramer (Table S1). It was noted that the molecular mass across the peak appeared to increase from the leading- to the trailing-edge of the peak, which is suggestive of non-ideal behaviour.

### 2.5. Analytical Ultracentrifugation

**Figure 4:**
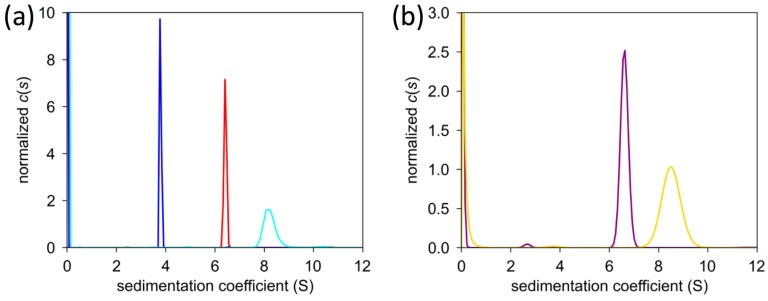
Sedimentation coefficient distribution for Vip3Ag4 at varying concentrations. (**a**) Interference data. Blue 1.0 mg/mL, red 0.5 mg/mL and cyan 0.25 mg/mL; (**b**) Absorbance data. Purple 0.5 mg/mL and yellow 0.25 mg/mL.

Analysis of Vip3Ag4 by sedimentation velocity (SV) also suggests that the protein is tetrameric (Table S2). During analysis of the interference data, corrections were made for buffer sedimentation, without which the molecular masses obtained were inconsistent with those obtained from analysis of the absorbance data and from the SEC-MALLS analysis. A decrease in sedimentation coefficient with increasing concentration was noted, suggestive of non-ideal sedimentation (Figure 4). An attempt to determine the concentration-dependent coefficient, k_s_, by linear extrapolation of a plot of 1/S_(20,w)_ against concentration yielded very high values, suggesting that other factors apart from excluded volume, such as charge, are contributing significantly to the sedimentation behaviour of this protein.

### 2.6. Surface Structure of Vip3Ag

**Figure 5:**
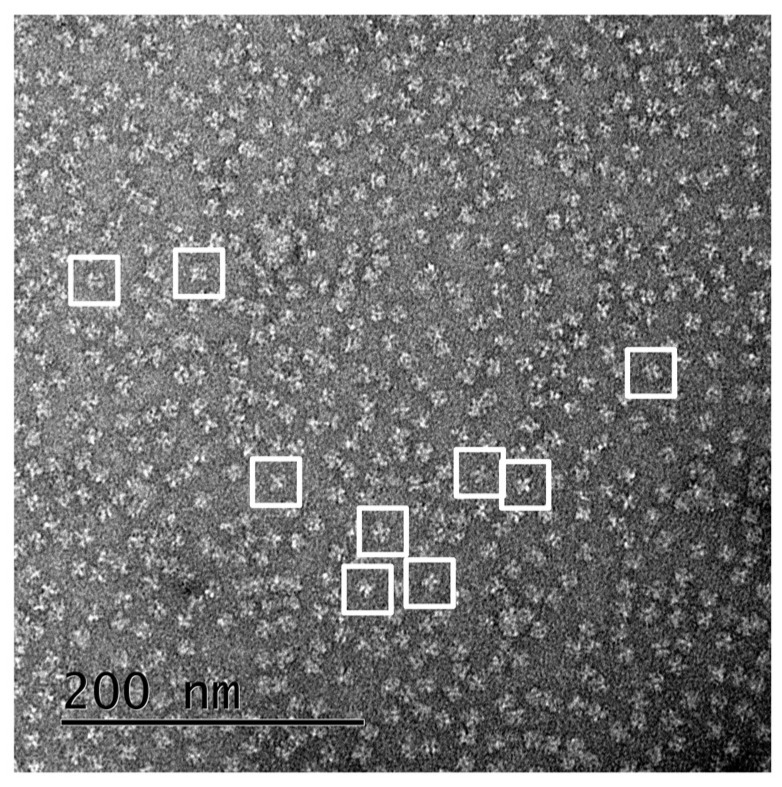
TEM grid. A representative example 40,000× TEM grid is shown with some example single particles captured in 128 × 128 pixel boxes.

**Figure 6:**
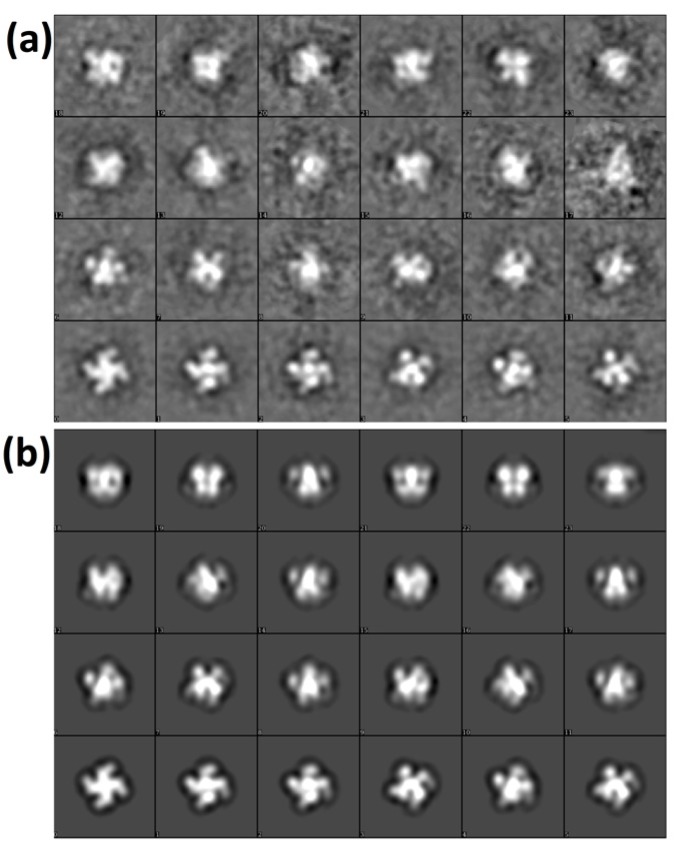
Vip3Ag4 TEM 2D class averages. (**a**) 2D-class averages for Vip3Ag4 particles; (**b**) reprojections from the final 3D model.

**Figure 7:**
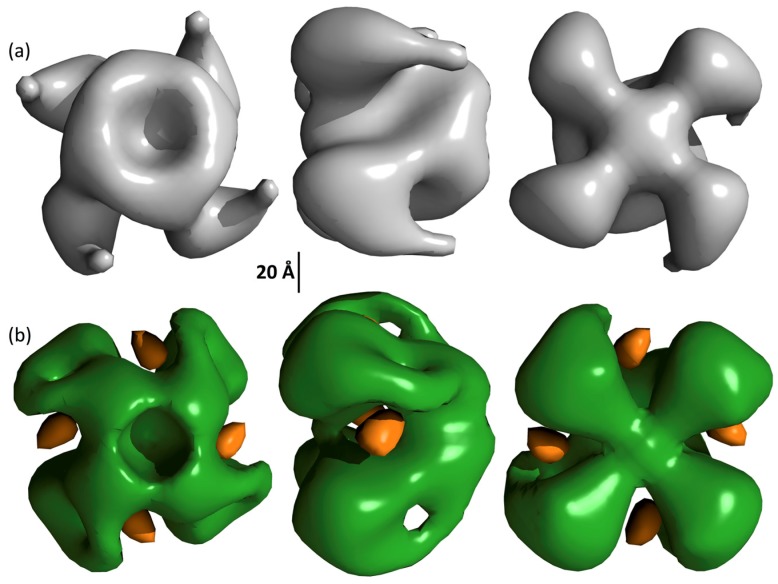
Surface topology of Vip3Ag4. Structures, with (**a**) and without (**b**) nanogold are displayed at volume shells corresponding to the expected molecular mass of Vip3 tetramers (380 kDa). The structure of the protein in the presence of gold is shown in green while the gold is shown in orange. Topology displayed using UCSF Chimera [28].

From 100 TEM images of grids incubated with Vip3Ag4, 3492 single particles were picked. Many of these appeared visually to show four-fold symmetry, consistent with our SEC-MALLS and analytical ultracentrifugation (AUC) data (Figure 5). From these particles, EMAN 2 was used to generate a collection of 70 2D class averages (example subset shown in Figure 6a). These were used in the subsequent construction of a modelled surface of the Vip3Ag4 protein (Figure 7a), based on four-fold symmetry as indicated from our SEC-MALLS and SV data. An excellent correlation was observed between 2D class averages and reprojections from the final structure (Figure 6a,b). Fourier shell correlation (FSC) analysis in EMAN2 of structures derived from odd- and even-numbered particles indicated a resolution of approximately 33 Å (FSC = 0.5 criterion: Figure S3). The structure, displayed at a volume threshold consistent with a protein particle of approximately 380 kDa (Figure 7a), shows four clear lobes. When the analysis was repeated in the presence of Ni-NTA-nanogold particles, particle sets that were similar to the original protein were produced (Figure S4a). Reconstruction from the TEM images obtained, based on the initial structure, again gave good correlations between these class averages and the reprojections from the final structure (Figure S4b). Gold particles are clearly visible between the lobes of the tetramer (Figure 7b), indicating binding to the His tags on the recombinant proteins in these locations.

Analysis of the surface topology of the trypsin-treated Vip3Ag4 again showed good correlation between TEM class averages and reprojections from the model (Figure S5). This structure shows a small region of disconnected density that is likely to be an artifact that may be caused by a bias in the views of the protein on the grid that were used to generate the model. The overall structure shows little change relative to the unprocessed protein (Figure S6) within the resolution of the models. This is consistent with the data above showing that the structure of the complex is maintained with little change following trypsin treatment. 

## 3. Conclusions 

These results illustrate for the first time structural data on a member of the Vip3 protein family, at the level of surface topology. Our data indicate that the predominant molecular species of Vip3Ag4 is a tetramer, and that the tetrameric form and general topology are retained after trypsin treatment. Trypsinised Vip3A is able to form pores in artificial bilayers and in *M. sexta* gut cells [12], and pore forming toxins frequently require multimerisation before or during membrane insertion. Whether the tetrameric form of Vip3Ag4 seen here forms a structural precursor to pore formation remains to be established. In addition to providing initial structural insight into the Vip3Ag4 protein, this work establishes that the Vip3Ag4 purity and monodispersity, in addition to the actual size of the tetrameric form, make this protein a candidate for full structural analysis using the emerging technique of cryo-electron microscopy.

## 4. Materials and Methods

### 4.1. E. coli Expression and Purification of Vip3Ag4 Protein

The Vip3Ag4 protein was first identified in a Spanish collection of Bt strains and cloned for recombinant expression [29]. Recombinant *E. coli* BL21(DE3) strain harbouring the *vip3Ag4* gene in the pET28b vector was pre-cultured overnight in an orbital shaker at 37 °C and 200 rpm in 2 × YT medium supplemented with 50 μg/mL kanamycin. A 1/25 dilution of this pre-culture into 500 mL 2× YT medium containing 50 μg/mL kanamycin was further incubated for 8 h at 37 °C with vigorous agitation (250 rpm). Protein expression was induced by adding isopropyl-beta-D-1-thiogalactopyranoside to a final concentration of 1 mM and incubation for up to 16 h. Samples were centrifuged at 5000 *g* for 15 min at 4 °C, and the resulting pellet was weighed and resuspended with 3 mL per gram of sonication buffer (20 mM sodium phosphate buffer pH 7.4, 0.5 M NaCl, 3 mg/mL lysozyme (Sigma-Aldrich, Gillingham, United Kingdom), 25 U Benzonase (Novagen, Madison, Wis, USA) and 100 μM phenyl-methylsulfonyl fluoride). Samples were further incubated at 37 °C under gentle agitation for 1 h and were sonicated on ice with a Branson analog sonifier 250 (Branson Ultrasonics Corporation, Danbury, CT, USA) by applying two 1 min pulses with a constant duty cycle at 60 W, separated by a 1 min cooling period. Insoluble material was pelleted at 12,000 *g* for 30 min at 4 °C and the soluble fraction was sequentially filtered through sterile 0.45 and 0.22 μm syringe filters. Protein purification was performed at room temperature (RT) using Protino Ni-TED 2000 Packed Columns following the manufacturer’s instructions (Macherey–Nagel, Düren, Germany). After the polyhistidine-tagged protein was eluted, a buffer exchange procedure was performed immediately with Milli-Q water and GE Healthcare PD-10 desalting columns to reduce protein aggregation and precipitation [29]. Subsequently, 5 mL samples were applied to a Hi-Prep 16/60 Sephacryl S-300 size exclusion column equilibrated with 20 mM TrisHCl, pH 8.0, 300 mM NaCl using an AKTA Prime Plus (GE Healthcare, Little Chalfont, UK) at a flow rate of 0.5 mL/min. The same column was also calibrated with gel filtration standards (BioRad, Watford, UK). Fractions (10 mL) were collected and the protein concentration was quantified by the Bradford method [30].

### 4.2. Trypsin Treatment of Vip3Ag4

Vip3Ag4 was diluted to 0.4 mg/mL in PBS and 200 µL was incubated with 8 µL of 0.1 mg/mL trypsin (a 100:1 *w:w* ratio) at 37 °C for 2 h. The products of the incubation were separated by SDS PAGE (12% acrylamide) with a tricine running buffer. Bands were blotted onto PVDF membrane and revealed by rapid staining with Coomassie blue R-250 and subjected to *N*‑terminal sequencing (Abingdon Health Laboratory Services, Birmingham, UK). A sample from the same digestion was also analysed by mass spectrometry (Waters, Elstree, UK, Tri‑wave IMS system with integrated high resolution MS capability).

### 4.3. Mass Spectrometry

Mass spectrometry was carried out using a Waters, Elstree, UK, Synapt G2-Si time of flight mass spectrometer coupled to a Waters Acquity UPLC equipped with a UPLC C4 column held at 60 °C. 

### 4.4. Circular Dichroism

Purified tetrameric Vip3Ag4 protein at 0.25 mg/m was desalted using a PD10 column (GE Healthcare) and analysed using a Chirascan^TM^ CD spectrometer (Applied Photophysics, Leatherhead, UK) at wavelengths between 400–180 nm at 0.5 nm intervals, compared to a sample of its original buffer (20 mM TrisHCl, pH 8.0, 300 mM NaCl) desalted in the same way, as a blank. Data were analysed using the Dichroweb server [31].

### 4.5. SEC-MALLS

A size exclusion chromatography—multi angle laser light scattering (SEC-MALLS) experiment was performed using a Superdex 200 10/300 Increase column (GE Healthcare) and an AktaPure 25 System (GE Healthcare). The Vip3Ag4 protein sample (100 μL), at a concentration of 1.0 mg/mL, was loaded onto the gel filtration column and eluted with one column volume (24 mL) of 20 mM TrisHCl, 300 mM NaCl buffer, pH 8.0 at a flow rate of 0.7 mL/min. The eluting protein was monitored using a DAWN HELEOS-II 18-angle light scattering detector (Wyatt Technologies, Santa Barbara, CA, USA) equipped with a WyattQELS dynamic light scattering module, a U9-M UV/Vis detector (GE Healthcare), and an Optilab T-rEX refractive index monitor (Wyatt Technologies). Data were analysed by using Astra (Wyatt Technologies) using the default refractive index increment value of 0.185 mL/g.

### 4.6. Analytical Ultracentrifugation

Sedimentation velocity (SV) experiments were conducted on a Beckman ProteomeLab XL-I analytical ultracentrifuge using an An-60 Ti rotor at 20 °C. Protein concentrations of 1.0, 0.5 and 0.25 mg/mL, in 20 mM Tris, 300 mM NaCl buffer, pH 8.0, were centrifuged at 20,000 rpm. Absorbance data, at 280 nm, and interference data were collected. Data were analysed using the program SEDFIT, fitting to the c(s) model. The density and viscosity of the buffer were measured using a DMA 5000 M densitometer (Anton-Paar, St Albans, UK) equipped with a Lovis 200 ME viscometer. The protein partial specific volume was calculated using SEDNTERP to allow for the calculation of molecular weight.

### 4.7. Transmission Electron Microscopy and Single Particle Analysis

Protein samples (100 μg/mL in Tris-HCl pH 8.0, 300 mM NaCl) were adsorbed on to carbon-coated 400 mesh copper grids and negatively stained with 2% (*w/v*) uranyl acetate. Transmission electron microscopy (TEM) images were recorded in the Cardiff University high-resolution TEM facility, using a Jeol JEM-2100 LaB_6_ transmission electron microscope operating at 200 kV, equipped with a 2 k Gatan Ultrascan camera, at a specimen level increment of 2.47 Å/pixel. EMAN 2 software [32] was used to process 3492 manually selected particles in 128 × 128 pixel boxes. The final model was generated using four-fold symmetry from a total of 14 iterations of refinement, and Fourier shell correlation (FSC) analysis of structures generated from even-and odd-numbered particles indicated a resolution of ~33 Å (FSC = 0.5 criterion).

For gold labelling, purified Vip3Ag4 was incubated for 1 min at room temperature with a 10:1 molar excess of 1.8 nm diameter nickel-nitrilotriacetic acid (Ni-NTA)-Nanogold particles (Nanoprobes, Yaphank, NY, USA). Samples were adsorbed onto grids and washed with Nanogold suspension to prevent unbinding of the protein-associated gold particles. After TEM and image processing, 6286 gold-labeled Vip3Ag4 particles (in 128 × 128 pixel boxes) were automatically selected. Three-dimensional structures were generated using the refined non-labelled Vip3Ag4 structure as a starting model with eight rounds of iterative refinement.

For analysis of the trypsin-treated Vip3Ag4, samples were processed as for untreated protein (above). Following the selection of 3543 particles, EMAN2 was used to construct a new model (without reference to the model for unprocessed protein). 

## Supplementary Materials

The following are available online at www.mdpi.com/2072-6651/9/5/165/s1, Figure S1: Sequence of the Vip3Ag4 protein. Bold letters represent the sequence added to the N-terminus from the expression construct and including the His-tag. Trypsin cut sites are shown as // above the two amino acids separated by the cleavage. Numbering is given for the full-length, naturally-occurring Vip3Ag4 sequence, Figure S2: CD analysis of Vip3Ag4. The trace for the 0.25 mg/ml Vip3Ag4 sample is shown between 185 and 240 nm. The green curve represents the experimental data, the blue curve the data generated from the reference set, and the pink lines show the difference between the experimental data and the reference data, Figure S3: Vip3Ag4 FSC curve. The curve for the 14th iteration of the EMAN model is shown and indicates a resolution of ~33 Å, Figure S4: Vip3Ag4 nanogold TEM 2D class averages. (a) 2D-class averages for Vip3Ag4 gold-labelled particles. (b) reprojections from the final 3D model, Figure S5: (a) Class averages (left) and 2D-reprojections (right) from the 3D model of trypsin-treated Vip3Ag4. (b) Fourier shell correlation (FSC) of 3D-structures derived from even- and odd-numbered particles indicating a resolution (FSC 0.5 criterion) of approximately 26Å, Figure S6: (a) 3 views of 3D-structure of trypsin-treated Vip3Ag4. A small region of disconnected density (likely to have resulted from some mis-classification of particles in 3D-reconstruction) is indicated with an arrow. (b) Comparison of trypsin-treated (cyan) and native Vip3Ag4 (green) single particle-derived structures, Table S1: Molar mass and hydrodynamic analysis of Vip3Ag4 determined by SEC-MALLS. Table S2: Best-fit parameters obtained from AUC SV data analysis.
