# Supplementary material for: The Vip3Ag4 Insecticidal Protoxin from Bacillus thuringiensis Adopts A Tetrameric Configuration That Is Maintained on Proteolysis
Source: Toxins (Basel). 2017 May 14;9(5):165. doi: 10.3390/toxins9050165 (PMC5450713; doi:10.3390/toxins9050165)
Supplement: Supplementary file 1 [file toxins-09-00165-s001.pdf]

Supplementary Materials: The Vip3Ag4 Insecticidal Protoxin from *Bacillus thuringiensis* Adopts A Tetrameric Configuration That Is Maintained on Proteolysis

Leopoldo Palma, David J. Scott, Gemma Harris, Salah-Ud Din, Thomas L. Williams, Oliver J. Roberts, Mark T. Young, Primitivo Caballero and Colin Berry

```
                                      //      6         16         26
MGSSHHHHHH SSGLVPRGSH MASMTGGQQM GRDPMNKNNT KLNARALPSF IDYFNGIYGF

        36         46         56         66         76         86
ATGIKDIMNM IFKTDTGGNL TLDEILKNQQ LLNEISGKLD GVNGSLNDLI AQGNLNTELS

        96        106        116        126        136        146
KEILKIANEQ NQVLNDVNNK LNAINTMLHI YLPKITSMLN DVMKQNYALS LQIEYLSKQL

       156        166        176      //186        196  //    206
QEISDKLDVI NVNVLINSTL TEITPAYQRM KYVNEKFEDL TFATETTLKV KKNSSPADIL

       216        226        236        246        256        266
DELTELTELA KSVTKNDVDG FEFYLNTFHD VMVGNNLFGR SALKTASELI AKENVKTSGS

       276        286        296        306        316        326
EVGNVYNFLI VLTALQAKAF LTLTTCRKLL GLADIDYTFI MNEHLDKEKE EFRVNILPTL

       336        346        356        366        376        386
SNTFSNPNYA KAKGSNEDAK IIVEAKPGYA LVGFEMSNDS ITVLKAYQAK LKQDYQVDKD

       396        406        416        426        436        446
SLSEIVYGDM DKLLCPDQSE QIYYTNNIAF PNEYVITKIT FTKKMNSLRY EATANFYDSS

       456        466        476        486        496        506
TGDIDLNKTK VESSEAEYST LSASTDGVYM PLGIISETFL TPINGFGIVV DENSKLVNLT

       516        526        536        546        556        566
CKSYLREVLL ATDLSNKETK LIVPPIGFIS NIVENGNLEG ENLEPWKANN KNAYVDHTGG

       576        586        596        606        616        626
VNGTKALYVH KDGEFSQFIG DKLKSKTEYV IQYIVKGKAS ILLKDEKNGD CIYEDTNNGL

       636        646        656        666        676        686
EDFQTITKSF ITGTDSSGVH LIFNSQNGDE AFGENFTISE IRLSEDLLSP ELINSDAWVG

       696        706        716        726        736        746
SQGTWISGNS LTINSNVNGT FRQNLSLESY STYSMNFNVN GFAKVTVRNS REVLFEKNYP

       756        766        776        786
QLSPKDISEK FTTAANNTGL YVELSRFTSG GAINFRNFSI K
```

Figure S1. Sequence of the Vip3Ag4 protein. Bold letters represent the sequence added to the N-terminus from the expression construct and including the His-tag. Trypsin cut sites are shown as // above the two amino acids separated by the cleavage. Numbering is given for the full-length, naturally-occurring Vip3Ag4 sequence.

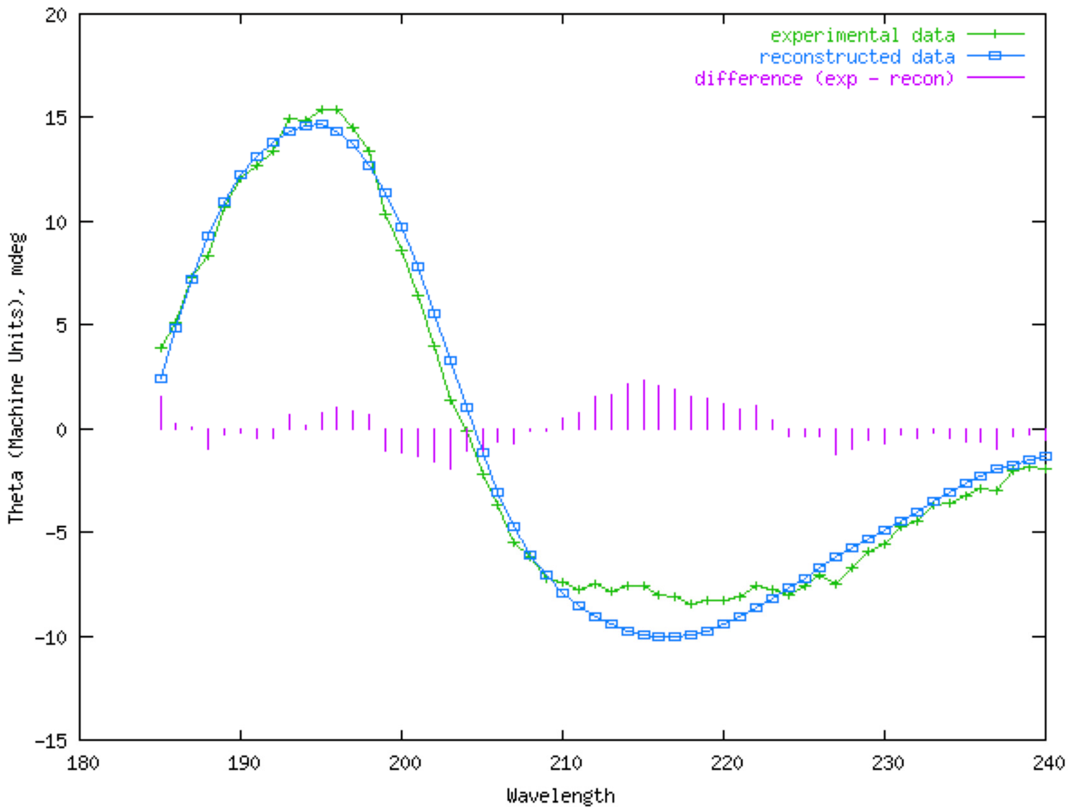

Figure S2. CD analysis of Vip3Ag4. The trace for the 0.25 mg/ml Vip3Ag4 sample is shown between 185 and 240 nm. The green curve represents the experimental data, the blue curve the data generated from the reference set, and the pink lines show the difference between the experimental data and the reference data.

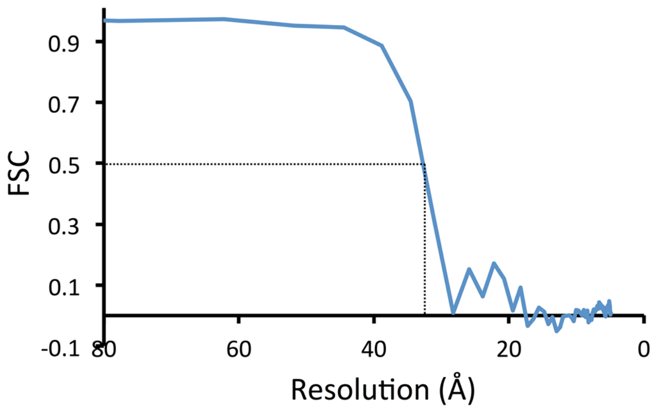

Resolution at FSC=0.5; 33 Å.

Figure S3. Vip3Ag4 FSC curve. The curve for the 14th iteration of the EMAN model is shown and indicates a resolution of ~33 Å.

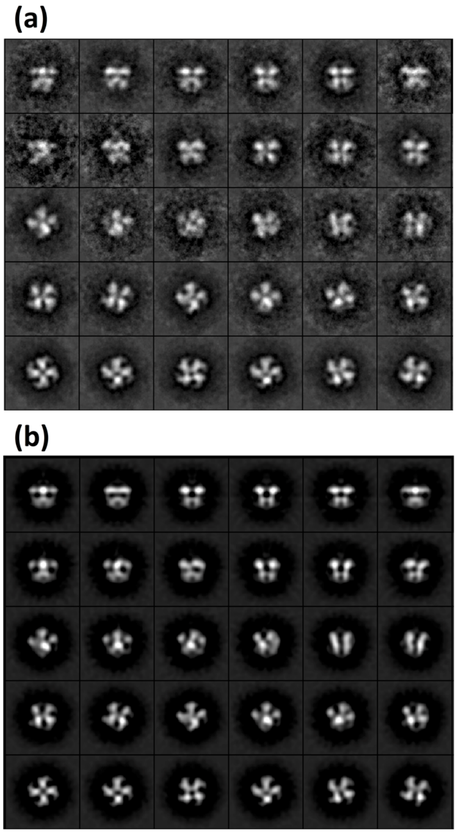

Figure S4. Vip3Ag4 nanogold TEM 2D class averages. (**a**) 2D-class averages for Vip3Ag4 gold-labelled particles. (**b**) reprojections from the final 3D model.

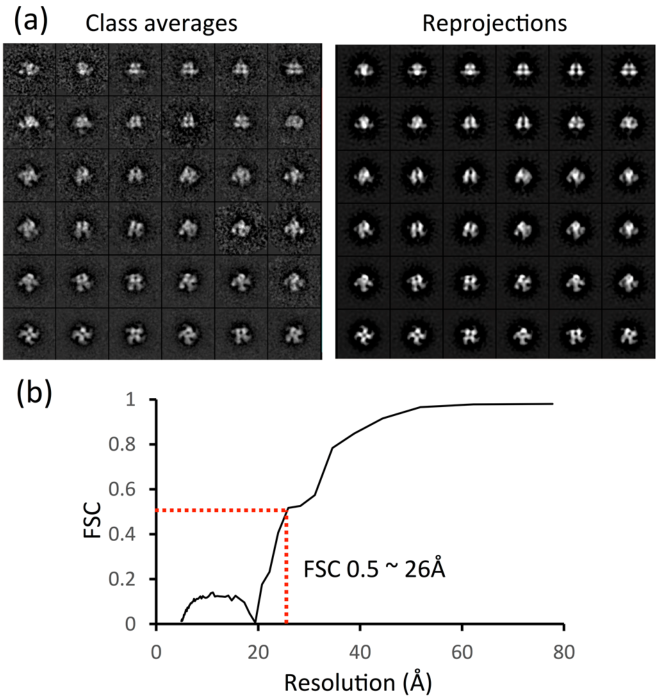

Figure S5. (**a**) Class averages (left) and 2D-reprojections (right) from the 3D model of trypsin-treated Vip3Ag4. (**b**) Fourier shell correlation (FSC) of 3D-structures derived from even- and odd-numbered particles indicating a resolution (FSC 0.5 criterion) of approximately 26Å.

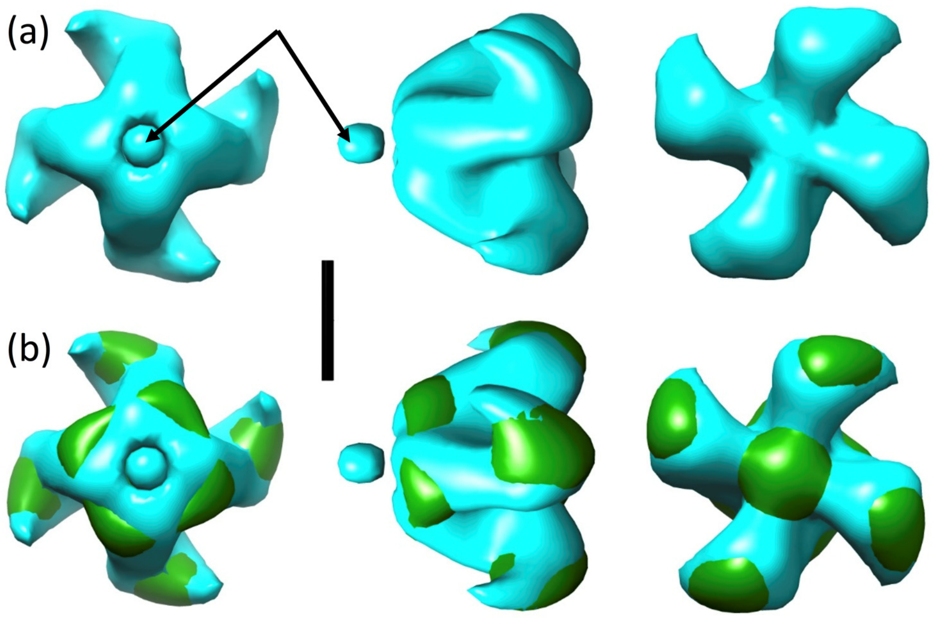

Figure S6. (**a**) 3 views of 3D-structure of trypsin-treated Vip3Ag4. A small region of disconnected density (likely to have resulted from some mis-classification of particles in 3D-reconstruction) is indicated with an arrow. (**b**) Comparison of trypsin-treated (cyan) and native Vip3Ag4 (green) single particle-derived structures.

Table S1. Molar mass and hydrodynamic analysis of Vip3Ag4 determined by SEC-MALLS.

Parameter	Vip3Ag4
Mn (kDa)	338 (± 0.1%)
Mw (kDa)	336 (± 0.1%)
Polydispersity (Mw/Mn)	1.006 (± 0.1%)
Rh(Q)z (nm)	6.9 (± 1.8%)
Rh(Q) (ave) (nm)	6.8 (± 0.3%)

Table S2. Best-fit parameters obtained from AUC SV data analysis.

**Parameter**	**Interference data**			**Absorbance data**		**Interference data without buffer correction**		
Concentration (mg/mL)	1.00	0.50	0.25	0.50	0.25	1.00	0.50	0.25
Fit rmsd	0.017	0.005	0.004	0.012	0.008	0.105	0.058	0.029
sw (S)	3.8	6.4	8.2	6.6	8.5	3.9	6.5	8.4
$sw_{(20,w)}$ (S)	4.1	6.9	8.8	7.1	9.1	4.1	6.8	8.8
Peak (% of total)	99.5	98.0	54.6	68.3	67.0	11.0	6.2	3.8
f/f_0	4.0	2.5	2.0	2.3	1.9	3.4	1.8	1.4
MW (kDa)	301	326	352	311	335	236	200	197
Stokes radius (nm)	17.6	11.3	9.5	10.4	8.7	13.8	7.0	5.3
a/b (oblate) (nm)	65.7	36.4	17.8	24.9	14.8	51.7	12.7	5.1
a/b (prolate) (nm)	74.9	33.6	15.6	22.1	13.0	52.0	11.2	4.8
